# Exploring Circulating Tumor DNA (CtDNA) and Its Role in Early Detection of Cancer: A Systematic Review

**DOI:** 10.7759/cureus.45784

**Published:** 2023-09-22

**Authors:** Parikshit Bittla, Simran Kaur, Vani Sojitra, Anam Zahra, Jhenelle Hutchinson, Oluwafolawemi Adefokun, Safeera Khan

**Affiliations:** 1 Internal Medicine, California Institute of Behavioral Neurosciences and Psychology, Fairfield, USA; 2 Surgery, California Institute of Behavioral Neurosciences and Psychology, Fairfield, USA

**Keywords:** prevention in primary care, medical screening, liquid biopsy, early detection of cancer, circulating tumor dna (ctdna)

## Abstract

There is a significant increase in the need for an efficient screening method that might identify cancer at an early stage and could improve patients' long-term survival due to the continued rise in cancer incidence and associated mortality. One such effort involved using circulating tumor DNA (ctDNA) as a rescue agent for a non-invasive blood test that may identify many tumors. A tumor marker called ctDNA is created by cells with the same DNA alterations. Due to its shorter half-life, it may be useful for both early cancer detection and real-time monitoring of tumor development, therapeutic response, and tumor outcomes. We obtained 156 papers from PUBMED using the MeSH approach in accordance with the Preferred Reporting Items for Systematic Review and Meta-Analysis (PRISMA) criteria and ten articles from additional online resources. After removing articles with irrelevant titles and screening the abstract and full text of the articles that contained information unrelated to or not specific to the title query using inclusion and exclusion criteria, 18 out of 166 articles were chosen for the quality check. Fourteen medium to high-quality papers were chosen out of the 18 publications to be included in the study design.

The reviewed literature showed no significant utility of ctDNA in detecting early-stage tumors of size less than 1 cm diameter. Still, the ideal screening test would require the assay to detect a size <5 mm tumor, which is nearly impossible with the current data. The sensitivity and specificity of the assay ranged from 69% to 98% and 99%, respectively. Furthermore, CancerSEEK achieves tumor origin localization in 83% of cases, while targeted error correction sequencing (TEC-Seq) assays demonstrate a cancer detection rate ranging from 59% to 71%, depending on the type of cancer. However, it could be of great value as a prognostic indicator, and the levels are associated with progression-free survival (PFS) and overall survival (OS) rates, wherein the positive detection of ctDNA is associated with worse OS compared to the tumors detected through standard procedures, with an odds ratio (OS) of 4.83. We conclude that ctDNA could be better applied in cancer patients for prognosis, disease progression monitoring, and treatment outcomes compared to its use in early cancer detection. Due to its specific feature of recognizing the tumor-related mutations, it could be implemented as a supplemental tool to assess the nature of the tumor, grade, and size of the tumor and for predicting the outcomes by pre-operative and post-operative evaluation of the tumor marker, ctDNA, and thereby estimating PFS and OS depending on the level of marker present. A vast amount of research is required in early detection to determine the sensitivity, specificity, false positive rates, and false negative rates in evaluating its true potential as a screening tool. Even if the test could detect the mutations, an extensive workup for the search of tumor is required as the assay could only detect but cannot localize the disease. Establishing the clinical validity and utility of ctDNA is imperative for its implementation in future clinical practice.

## Introduction and background

"You can be a victim of cancer or a survivor of cancer. It is a mindset," as said by Dave Pelzer. There has been a tremendous increase in the incidence of cancers, with 23.6 million new global cancer cases in 2019, causing 10.0 million cancer deaths and an estimated 250 million disability-adjusted life years (DALYs) due to cancer [1]. Experts predict that by 2040, the continued adaptation of unhealthy lifestyles and aging populations will result in an expected cancer incidence of 30.2 million cases, leading to 16.3 million deaths. According to The United Nations (UN) Sustainable Development Goals (SDGs), the cancer burden has to be reduced as a part of target 3.4 - " BY 2030, reduce one-third of premature mortality by noncommunicable diseases (NCDs) through prevention and treatment," suggesting an increased need for cancer prevention and reduction in cancer burden [1-3].

With an increasing delay between symptom onset and treatment, chances of survival progressively decline, making screening an important tool for early detection and improving survival [4,5]. Currently, population-based screening for four common cancers in high-income countries leads to a reduction in the incidence of breast cancer, cervical cancer, prostate cancer, and colorectal cancer [4,6-9]. With no proper screening, 50% of lung, colorectal, cervical, ovarian, and pancreatic cancers, 30% of breast cancers, and 20% of prostate cancers are diagnosed later when treatment options are limited and less effective [10,11].

The World Health Organization (WHO) has defined cancer screening as the "ability to identify pre-clinical or a pre-cancerous lesion in a healthy population" [10,12]. Currently, The US Preventive Services Task Force (USPTF) recommends a single screening tool for breast (mammography), colon (colonoscopy), cervical (pap smear), lung (low-dose computed tomography (LDCT)), and prostate (blood-based prostate-specific antigen (PSA)) in age-specific at-risk population as an individual decision [10,13-15]. Additional population-specific screening strategies have been described for individuals at risk by experts for anal, esophageal, gastric, and hepatobiliary cancers [10,16-17]. There are many novel screening tests developed recently, such as Breath Biopsy for breast and lung cancer, Cytosponge for esophageal cancer, human papillomavirus (HPV) testing for cervical cancer, methylation detection, and genome-wide-DNA fragmentation pattern [18-23]. As screening strongly relies on adherence rate, the proportion of individuals undergoing screening is a long-lasting challenge, and there is a current trend of using mHealth technologies for improving the screening uptake by the public through SMS text messages and telephone calls, which gained high acceptance among the population [4].

Although the benefits of cancer screening are well understood, there is a potential for harm that comes with screening, including false-positive screening leading to additional testing and its complications of over-diagnosis and over-treatment impacting the psychological and social aspects of the screening population [10]. This prompts us to develop a highly sensitive, non-invasive screening test that could detect multiple cancers. This was made possible by discovering circulating tumor DNA (ctDNA) in 1948 for the first time in healthy individuals [24].

Cell-free DNA fragments are released into circulation by tumor cells with an approximate length of 166, 332, or 498 base pairs exhibiting tumor-specific alterations with a half-life of 114 minutes. CtDNA is a class of circulating free DNA (cfDNA), with fragments of tumor cell DNA containing the same gene defects as the tumor DNA. Because of its short life, ctDNA levels can provide an index of real-time dynamic changes in tumors. Due to the possibility of real-time monitoring with specific tumor DNA mutations and its non-invasive nature allowing simple and repeated monitoring, ctDNA can potentially replace conventional tissue biopsies for diagnosis and is considered a liquid biopsy marker [25-26].

Currently, research is evaluating the potential of ctDNA as a marker of minimal residual disease for curative purposes and as a screening tool in early cancer detection. In clinical practice, it is already being used to guide systemic therapy in metastatic disease, thereby changing the approach to oncology [26]. Recent researches prove the utility of cfDNA in the identification of fetal genetic anomalies, solid organ transplant, and identification of tumor mutations [27-29]. Initially, ctDNA was used to assess tumor burden, where it was present among cfDNA at variant allele fraction (VAFs) of <0.1% to 10%, representing the percentage of mutant DNA compared to normal DNA. Higher numbers of VAFs were detected in patients with higher tumor burden and worse prognosis [26,30-31].

The current systematic review evaluates the role of ctDNA in early cancer detection and its implementation in current screening tools, aiming to avoid conventional invasive procedures. Additionally, we aimed to assess the impact of standardizing ctDNA as a sole strategy for detecting multiple tumors using the simple and non-invasive technique.

## Review

Methods and search strategy

We strictly followed the guidelines of Preferred Reporting Items for Systematic Reviews and Meta-Analysis (PRISMA) [32]. We comprehensively searched different electronic databases for relevant literature on ctDNA and its prognostic value in early cancer detection. PubMed, PubMed Central, and MEDLINE were the major databases used to search appropriate keywords and Medical Subject Headings (MeSH) for finding relevant articles. The following keywords were used in the literature search: "ctDNA," "circulating tumor DNA," "cancer screening," and "early cancer detection."

The final MeSH strategy for PubMed is as follows: ("Circulating Tumor DNA/analysis"[Majr] OR "Circulating Tumor DNA/blood"[Majr] OR "Circulating Tumor DNA/isolation and purification"[Majr] OR "Circulating Tumor DNA/metabolism"[Majr] OR "Circulating Tumor DNA/physiology"[Majr]) AND ("Early Detection of Cancer/methods"[Mesh] OR "Early Detection of Cancer/mortality"[Mesh] OR "Early Detection of Cancer/trends"[Mesh]).

After we got relevant articles through the PubMed Advanced search and other databases, they were transferred into Endnote. We exported the papers from the Endnote to Excel. Two personnel reviewed the search results independently. We screened the titles of each paper and excluded those with unrelated topics or topics focusing on specific cancers. After carefully reviewing the abstracts and full text of the articles, we selected papers for a quality check using standardized quality assessment tools specific to each study. Papers with low quality were then excluded from further consideration.

Inclusion and Exclusion Criteria

We included observational studies, randomized control trials (RCTs), systematic reviews, traditional reviews, meta-analysis journals, and other articles in English. We included studies carried out after 2013 on older individuals at risk for cancer development and excluded editorials, perspectives, case reports, peer reviews, grey literature, unpublished studies, and animal studies (Table 1).

**Table 1:** Inclusion and exclusion criteria RCTs, randomized controlled trials

	Inclusion Criteria	Exclusion Criteria
Language	Literature published in the English language	Literature published in languages other than English
Type of Study	observational studies, RCTs, systematic reviews, traditional reviews, meta-analysis journals	Editorials, perspectives, case reports, peer reviews, grey literature, unpublished studies, and animal studies
Year of Publishing	Articles published after 2013	Articles published before 2013
Content of Study	Articles with content relevant to the research question	Articles focusing on specific cancers or articles irrelevant to the research question
Age of Individuals	Individuals with age 18 years or above	Individuals aged <18 years

Quality Appraisal of Studies and Bias Assessment

**Figure 1:**
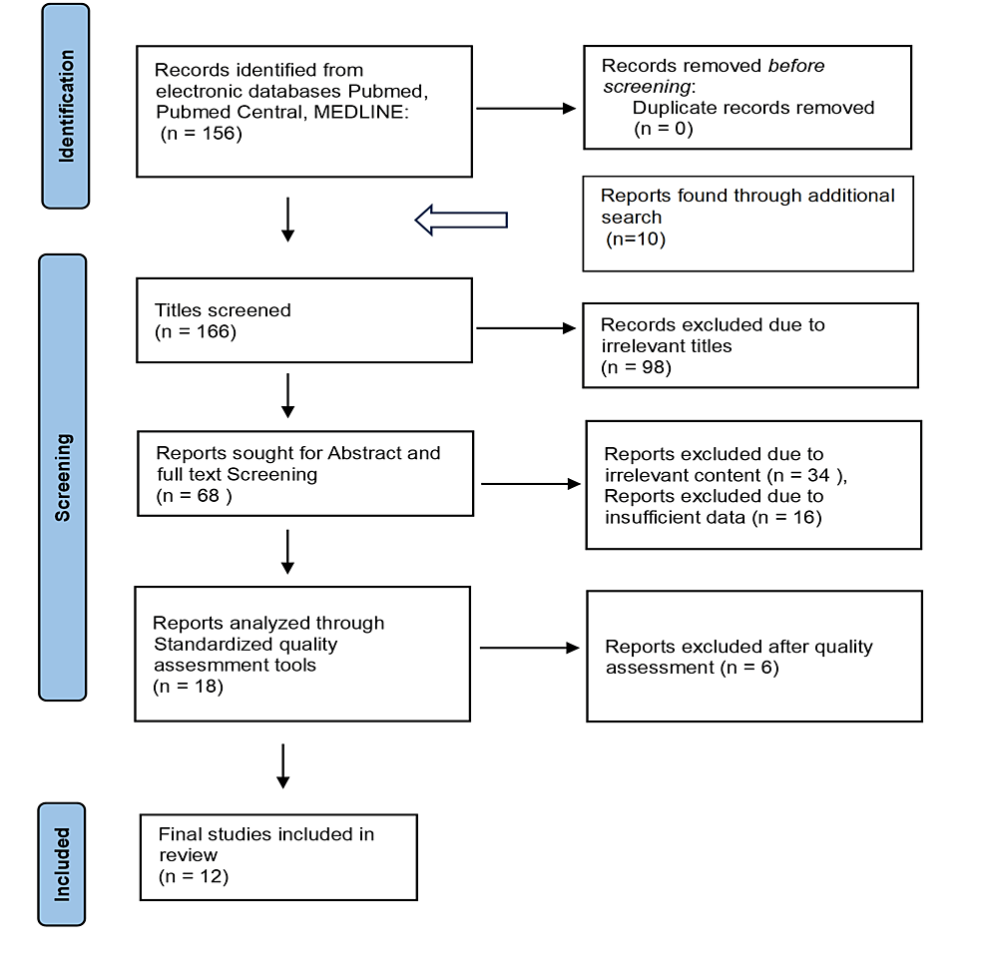
Preferred Reported Items for Systematic Review and Meta-Analysis (PRISMA) flowchart PRISMA, Preferred Reported Items for Systematic Review and Meta-Analysis

We finalized 18 articles for quality assessment after excluding irrelevant titles and screening through the abstract and full text of the articles recruited via an electronic database. Of the 18 articles, 12 were categorized as high or medium-quality papers and included in the study (Figure 1). The tools used for the analysis of quality include the Assessment of Multiple Systematic Reviews (AMSTAR) criteria for systematic review and meta-analysis (Table 2), the New Castle - Ottawa scale for observational studies (Table 3 and Table 4), the Scale for the Assessment of Narrative Review Articles (SANRA) criteria for traditional reviews (Table 5).

**Table 2:** AMSTAR criteria AMSTAR, A Measurement Tool to Assess Systematic Reviews

AMSTAR Criteria	IA Cree et al., 2017 [33]	JD Merker et al., 2018 [34]	Ocana A et al., 2016 [35]
Priori design provided	YES	YES	YES
Duplicate study selection, data extraction present	YES	YES	YES
Comprehensive literature search performed	YES	YES	YES
Was the status of publication used as inclusion criteria	YES	YES	YES
A list of inclusion and exclusion studies provided	YES	NO	YES
Characteristics of inclusion studies provided	YES	YES	YES
Quality of inclusion studies included and documented	NO	NO	NO
Quality of inclusion studies used appropriately in forming conclusions	NO	NO	NO
Appropriate methods used to combine studies	YES	YES	YES
Likelihood of publication bias assessed	NO	YES	YES
Conflict of interest included	YES	YES	NO
The final score assigned	8/11	8/11	8/11

**Table 3:** New Castle - Ottawa scale for longitudinal studies

New Castle - Ottawa Criteria	X Chen et al., 2021 [36]	X Chen et al., 2020 [37]
SELECTION		
Adequate case definition	1	1
Representativeness of cases	1	1
Selection of controls	0	1
Definition of controls	1	1
COMPARABILITY	2	2
EXPOSURE		
Ascertainment of exposure	1	1
Method of assessment for cases and controls	1	1
Non-response rate	1	1
Assigned score	8/9	8/9

**Table 4:** New Castle - Ottawa scale for cross-sectional studies

New Castle - Ottawa Scale	Schwaederle et al., 2022 [38]
SELECTION	
Representativeness of cases	1
Sample size	0
Non-response rate	1
Ascertainment of screening/surveillance tool	2
COMPARIBILITY	0
OUTCOME	
Assessment of outcome	2
Statistical test	1
ASSIGNED SCORE	7/9

**Table 5:** SANRA checklist SANRA, Scale for the Assessment of Narrative Review Articles

SANRA Checklist	J Phallen et al., 2017 [39]	JD Cohen et al., 2018 [40]	C Fiala et al., 2018 [41]	M Scarlotta et al., 2019 [42]	AH Ren et al., 2020 [43]	Dang DK et al., 2022 [26]
Justification of the articles' importance	2	2	2	2	2	2
Concrete aims/formulation of question	2	2	2	2	2	2
Description of literature search	0	0	1	1	1	0
Referencing of key statements	2	2	2	2	2	2
Scientific reasoning/appropriate evidence	2	2	2	2	2	2
Appropriate presentation of data	2	2	2	2	2	2
Assigned score	10/12	10/12	10/12	11/12	11/12	10/12

Results

The electronic database search yielded 166 articles as part of the search strategy. We conducted title screening and excluded 98 articles with irrelevant titles, leaving us with 68 articles for further screening. These remaining articles underwent abstract and full-text review, resulting in the selection of 18 articles for quality assessment. In conclusion, we included 12 high-quality articles in the study after identifying them through the final selection process [26,33-43]. The finalized articles comprise two case-control studies, one cross-sectional observational study, three systematic reviews, and six traditional reviews with 25,774 patients (Table 6). The results showed no significant utility of ctDNA in detecting early-stage tumors with less than 1 cm diameter. The sensitivity and specificity of the assay ranged from 69% to 98% and 99%, respectively.

**Table 6:** Characteristics of included studies ctDNA, circulating tumor DNA; OS, overall survival; MCED, multi-cancer early detection; TEC-Seq, targeted error correction sequencing

Author of the Study	Year of Publication	Type of Publication	Aim of the Study	Conclusion
Dang DK et al. [26]	2022	Traditional review	To determine the role of ctDNA as a cancer screening tool	CtDNA could be better used as a supplemental tool, which could help guide the management of tumors and their prognosis
IA Cree et al. [33]	2017	Systematic review	To identify a blood-based tumor marker for non-invasive cancer detection	ctDNA has the potential to be considered as a potential biomarker for cancer detection in future
JD Merker et al. [34]	2018	Systematic review	Utility of ctDNA in solid tumors	No strong evidence is available on the utility of ctNA in clinical practice.
Ocana A et al. [35]	2016	Systematic review	Association between ctDNA and overall survival in solid tumors	Positive ctDNA assay is associated with worse outcomes
X Chen et al. [36]	2021	Case-control study	To determine OS in patients who tested positive for MCED study	The outcome of patients with negative MCED was better when compared to those with a positive MCED
X Chen et al. [37]	2020	Longitudinal study	Detection of ctDNA using PanSeer, a non-invasive blood test four years before conventional tumor diagnosis	PanSeer could successfully detect cancer in asymptomatic individuals four years earlier than standard screening or diagnostic techniques
Schwaederle M et al. [38]	2016	Cross-sectional study	The ability of ctDNA to detect tumor mutations and its concordance with tissue biopsies	The ability of ctDNA to determine treatment and prognosis
J Phallen et al. [39]	2017	Clinical evaluation study	Evaluating the ability of the TEC-Seq technique to detect cfDNA	The test is useful in detecting early-stage cancers and assists in determining prognosis
JD Cohen et al. [40]	2018	Clinical evaluation study	Evaluating the ability of CancerSEEK, a multi-analyte blood test in tumor detection	CancerSEEK was successful in identifying and locating the majority of the tumors
C Fiala et al. [41]	2018	Traditional review	Applications of ctDNA in different aspects of cancer management focusing on early cancer detection	The sensitivity and specificity of ctDNA in tumor detection are fairly low and are not advisable to implement at the level of clinical practice
M Scarlotta et al. [42]	2019	Traditional review	Applications of the biomarkers - ctDNA, tumor cells, extracellular vesicles in the management of tumor	All three techniques showed promising results, while ctDNA was superior
AH Ren et al. [43]	2020	Traditional Review	Limitations of ctDNA as a tumor marker	The limitations of ctDNA are high enough to prompt further research for its implementation in routine care

Additionally, CancerSEEK achieves tumor origin localization in 83% of cases, while TEC-Seq assays demonstrate a cancer detection rate ranging from 59% to 71%, depending on the type of cancer. However, it could be of great value as a prognostic indicator, and the levels are associated with progression free survival (PFS) and overall survival (OS) rates, wherein the positive detection of ctDNA is associated with worse OS compared to the tumors detected through standard procedures, with an odds ratio (OR) of 4.83. Furthermore, the assay would be of tremendous use in predicting tumor outcomes. The utilities of ctDNA assay could be extended to monitor the disease progression and the treatment response as the levels correspond to tumor burden, metastasis, grade, and size.

Discussion

CtDNA and Its Biologic Characteristics

CtDNA is an excretory product released from the dead tumor cells into the bloodstream, with a genetic structure similar to the tumor cell. It could become a potential tumor marker in future clinical practice. cfDNA is released from the tumor cell with both nuclear and mitochondrial DNA made up of 160bp, and ctDNA is a fraction present in cfDNA, which consists of mutations that are highly specific to tumor DNA and is detected by polymerase chain reaction (PCR) assay OR digital droplet PCR or BEAMing assays. Its half-life varies from minutes to hours, representing the tumor's current status, and allowing "real-time assessment" of tumor biologics [42].

Different approaches have been described for detecting ctDNA, including TEC-Seq by J. Phallen et al., to detect known alterations specific to a particular type of cancer using next-generation sequencing (NGS) and parallel sequencing, which is claimed to have an error rate of <1 false positive rate per three million base sequences [39]. Furthermore, another technique called CancerSEEK, a new blood test, was proposed by J.D. Cohen et al. that could detect the eight most common cancers, namely lung, colorectal, ovarian, breast, esophageal, liver, pancreas, ovary, and stomach, with sensitivity for the later five of them ranging from 69% to 98% and specificity being more than 99% [40].

Most studies suggested using a minimum of 10 mL blood draw sample for analyzing the presence of ctDNA. Dang DK et al. suggested that plasma samples were better than serum samples and must be processed within hours of collection, as the delay would result in lysis of white blood cells (WBC), which could falsely elevate the amount of tumor DNA in the sample. The tube should be stored at -80°C [26]. The requirements for collecting a sample of ctDNA include the following: i) the parent tube must contain a volume of at least 3 mL, ii) the time period between sample collection and plasma isolation should be less than five days, and iii) the tube must have the plasma graded by either optical density or visualization [36-37].

The optimal specimen is the sample taken from plasma in lavender top tubes, stabilized in K2EDTA, and processed within six hours after collecting. The sample should undergo either filtration or sequential centrifugation at low and high speeds. Deviation from the above-mentioned conditions would lead to a falsely elevated level of ctDNA, particularly when the tube is stored for more than three to five days or rewarmed to 40°C. The study also mentioned that ctDNA levels might be influenced by certain conditions like smoking, pregnancy, heart diseases, inflammation, physical activity, and even diurnal variations [34]. ctDNA samples can also be obtained through cerebrospinal fluid (CSF) and urine, provided aerosolized DNA does not contaminate the sample [26].

Researchers commonly analyze ctDNA for methylation sequences, frequently present in tumor genes, and can be compared to regular plasma sequences. The detection of mutations in ctDNA is achieved using PCR-based assays. JD Merker et al. categorized ctDNA assays into two categories: targeted assays using real-time or digital PCR for known somatic mutations and the second broad coverage assay using NGS, detecting multiple genes. The two assays are not interchangeable because of the varying performances of individual categories [34].

CtDNA is present as variant allele fractions (VAFs) in cfDNA, with the levels varying from <0.1 to 10%, representing the ratio between mutated and total DNA. Available data from various studies showed that levels of VAFs increase with the increasing tumor burden. Hence, higher VAFs in DNA are a poor prognostic indicator associated with worse outcomes. The levels of cfDNA and ctDNA may vary with therapeutic intervention and respond to local or systemic therapy accordingly [26].

CtDNA as an Early-Stage Marker for Cancer Detection

As ctDNA has the same mutations as tumor DNA and is secreted into the bloodstream, there is an increasing hype in modern medicine to consider it as the biomarker for tumor detection and develop the necessary techniques needed for incorporation into clinical practice, substituting the cancer-specific screening tools with a single test to detect a wide range of cancers. However, this would eventually require tumor localization with existing tests for specific cancers, increasing the cost of detection and delayed diagnosis. The first milestone was contributed by the Food and Drug Administration (FDA) approval of the first blood-based cancer screening for colorectal cancer by using septin-9 methylation assay [33].

J Phallen et al., in the year of 2017, conducted a study to detect early-stage cancers using ctDNA through the technique of targeted error correction sequencing called TEC-seq for ultrasensitive detection of changes in the sequences of cfDNA and analyzed the plasma of 44 healthy individuals. The results showed that TEC-seq has a very low false positivity rate of one in three million sequences. Subsequently, when the plasma of 200 patients with common cancers such as breast, lung, colorectal, and ovarian was evaluated, it was able to detect mutations in 59% to 71% of patients depending on the type of cancer in the early stages like stage one and two, with the average level of cfDNA in plasma being 29 ng/mL (levels of cfDNA in the healthy individual were estimated to be nearly 7 ng/mL and in the patient with stage IV cancer to be around 66 ng/mL). However, the main challenge for early detection was identifying the de novo somatic mutations of tumor cells. The study addressed clonal hematopoiesis as a major confounding factor for tumor-specific mutations, which could lead to overdiagnosis and an increase in the false positive rate. Given the ability of ctDNA to detect cancer in nearly three-fourths of patients of the above-described tumors, the study concluded that positive results with ctDNA have to be followed with additional testing by imaging and other diagnostic modalities to confirm the presence of tumors and to identify the tumor of origin [39].

The study conducted by C Fiala et al. in 2018 was in concordance with the study mentioned above, reporting that ctDNA released from the tumor cells would be ten times more when compared with a normal cell, wherein the levels would range from 1 to 10 ng/mL in a healthy individual. As already described, the sensitivity and specificity would reach 100% even by detecting one mutation out of 50-500 or more cancer-associated mutations. Such detection would not be made possible with a conventional 10 mL blood draw sample when the tumor DNA level drops below 0.01%, leading to impossible detection of tumors. However, the definitive screening test would be able to detect the tumor of around 5 mm for breast cancer, corresponding to an early-stage tumor undetectable with mammography, making it impossible for detection with ctDNA assay. The convenient size of the tumor to be detected by ctDNA assay would range from 1 to 3 cm in diameter [41]. It, hence, could be used as a great supplemental detection tool, supported by the data from a study by Newman et al., where a deep sequencing approach detected EGFR kinase mutations in a 25,000-fold level of normal DNA. However, even at these ratios, the tumor would range from 0.1 to 1 g, which is well above the range for cancer detection.

Similar to the data provided by AH Ren et al., the above study also mentioned that even with a sensitivity of 100% and a specificity reaching 99.9%, the test would have a positive predictive value (PPV) of 20%, where 100 samples will be identified as false positives out of 100,000 samples. In another study done by Genovese et al., following 12,380 healthy individuals for two to seven years after an initial assessment of mutations in the samples provided identified clonal hematopoiesis as a confounding factor that increased false positive rates. 10% of the individuals aged above 65% were falsely identified by ctDNA. Around 42% of individuals identified as positive were diagnosed with clonal hematopoiesis in the next six months, lowering the sensitivity and reliability.

To overcome the limitations mentioned above for early detection at a smaller tumor size and to identify the source of mutations, JD Cohen et al. designed a multianalyte blood test in 2018 with 1005 patients with cancer of stages I to III, with two components (genetic alterations and protein biomarkers) named CancerSEEK, which could detect nearly eight types of cancers at an early stage. The test claimed to detect up to five types of cancers with a sensitivity of 69% to 98% and a specificity of nearly 99%, which correctly identified 70% of samples from cancer patients as positive and determined the anatomic location of the tumor in 83% case with localization being highest for colorectal and lowest for lung cancers. CancerSEEK could successfully identify at least one mutation sequence in 82% of cases and ≥ two mutations in 55% of cases, out of a study population of 805, with detection capability being highest for ovarian cancer (100%) and lowest for liver cancer (60%) [40].

The statistics showed that the sensitivity of CancerSEEK is highest for ovarian cancer, at 98%, and lowest for breast cancer, at 33%. The median sensitivity ranged from 73% to 78% in stages II and III cancers, respectively, which fell dramatically to a median of 43% in stage I cancers (highest for liver cancer with 100% and lowest for esophageal cancer with 20%). The specificity was identified to be greater than 99%, with only seven false positive cases out of 812 healthy individuals. The results were confirmed by analyzing the tumor-specific markers in the tumor tissue from 153 individuals with detectable ctDNA and comparing them with the results of CancerSEEK. The concordance rate was as high as 90%, with 138 individuals being positive for an identical tumor mutation, ranging from 100% in ovarian cancer to 82% in gastric cancer. Though the results seemed favorable for most cancer types, the test ran each base thousands of times to detect mutations with low prevalence, which was neither time nor cost-sensitive. However, after considering the confounding factors of inflammatory diseases and other conditions that could lead to a higher detection rate, the corrected sensitivity was estimated to be 55% for all eight cancer types. Interestingly, this correction would not change the sensitivity considerably for gastrointestinal and ovarian cancers due to the absence of defined screening tests for individuals with high risk. After a thorough evaluation of the following data, it is required that following a positive ctDNA assay, the individual has to be evaluated with conventional markers such as CA125, CA 19-9, CEA, or as specified for the tumor type [40].

However, in contrast to the results published on CancerSEEK, another study by JD Merker et al. in the same year revealed an opposite result. According to the data published, ctDNA assays failed to show concordance with the tissue samples, which could be attributed to many confounding factors such as tumor heterogeneity, tumor size, stage, sampling errors, and clonal hematopoiesis. One such example would be CNS tumors wherein the levels of ctDNA are substantially low due to the presence of the blood-brain barrier (BBB). Though the analytical specificity for ctDNA assays was >95%, evidence was lacking on clinical validity and utility in clinical practice outside the trials, which is yet to be determined by extensive research [34].

In 2019, M Scarlotta et al. described the Safe-Seq technique aided by BEAMing in patients with early localized tumors. It detected ctDNA in nearly 47% of patients with stage I and 86-100% with metastatic disease. The results support that ctDNA levels vary with tumor burden, and higher levels are detected in metastatic disease compared to a localized tumor. However, the detection rate through Safe-Seq was not high enough to accept ctDNA as a screening tool for early tumors. It could detect a later-stage tumor with greater sensitivity, which would be already symptomatic when ctDNA reaches the detection level and hence cannot be employed for screening strategies. The study also described another biomarker, circulating tumor cells (CTCs), for cancer detection, which identified lung cancers as benign lesions with 89% sensitivity and 100% specificity and could differentiate between patients of hepatocellular carcinoma (HCC) and healthy individuals with a sensitivity and specificity of 73%, and 93.4%, respectively, which is not sufficient enough to be used as a screening tool in clinical practice [42].

However, a longitudinal study done in 2020 by X Chen et al. demonstrated that a single non-invasive blood test named PanSeer, based on detecting methylation sequences in ctDNA, can detect the tumor in 95% of the asymptomatic population, who were later diagnosed as having cancer, with a confidence interval of 89-98%. As a part of the study, plasma samples from 123,115 healthy individuals were procured, and the tests were run on the samples for ctDNA methylated sequences. According to the study results, the test accurately identified the patients already diagnosed, with an overall sensitivity of 88% and specificity of 96%. It also detected cancer in 143 asymptomatic individuals diagnosed within four years. This suggests that the test could identify the cancer growth well before symptomatic or incidental presentation, undetectable to other measures [37].

According to the results published in the same year by AH Ren et al. in 2020, cancer detection is possible through ctDNA, with a sensitivity of 50% to 70% depending on the type of cancer and a specificity of 90% to 98%. Though the reported sensitivities and specificities seem to be high, again, the numbers are not enough to be implemented for screening relatively rare types of cancers, like pancreatic cancer. The calculations provided in the study show that with a specificity of 95%, even with a high sensitivity of 100%, the PPV (the percent of the population being truly positive with positive test results) is as low as 5%, and hence would result in a tremendous amount of false positives needing additional testing increasing the cost in search of tumor that is not present in the first place, creating anxiety in the patient. The technique also suffers from a high false negative ratio for tumors less than 10 mm due to inadequate levels secreted into the bloodstream and incorrect sampling and storing [43].

The inability of ctDNA to be used as a screening marker was supported by the experimental data published by GRAIL company, which termed the claimed sensitivity of ctDNA as an "overestimation." The data showed that the sensitivity of detection is only 10% in asymptomatic breast cancers detected by mammography compared to a sensitivity of 50% in clinically detectable breast cancers, which is a significant drop of fivefold. The team conducted another study to validate the sensitivity and collected samples from a prospective RCT for a cancer screening trial of prostate, lung, colorectal, and ovarian (PLCO) in men and women in the age range of 55 to 74 to assess the primary and secondary endpoints of screening. They selected 180 ovarian cancer samples as cases and 660 healthy samples as controls, and none of the biomarkers, such as cfDNA, could be proved as effective along with the conventional marker of CA125, showing a sensitivity of less than 50% in asymptomatic individuals.

Dang DK et al. published a study in 2022 supporting the above results. He concluded that for ctDNA to be used in the screening procedure, it should demonstrate a high clinical utility, which still needs to be assessed by clinical researchers. Also, a screening test must have a high negative predictive value, which is yet to be established for ctDNA as a tumor marker, and hence, its absence cannot rule out the disease. Currently, the PPV of the study still needs to be clinically validated. In light of the above facts, the study described ctDNA as a "companion diagnostic" instead of considering it as a sole screening assay [26].

Owing to the vast statistical data presented to date, the value of ctDNA for early diagnostic purposes is considered to be used only for adjunctive purposes combined with conventional evaluations for confirmation and localization. It cannot be used as a substitute for standard screening surveys.

CtDNA as a Prognostic Indicator and Marker of Surveillance

Although ctDNA cannot be relied on for the intended diagnostic purposes, it has shown tremendous utility as a predictive tool and could be used for cancer surveillance. The treatment protocol can be adjusted according to the quantitative levels of ctDNA, wherein the levels will decrease with appropriate treatment response and increase with disease progression; nevertheless, decreased sensitivity of ctDNA assay when done while on the treatment protocol poses the main challenge. Furthermore, the assay could detect resistant mutations earlier than standard radiographic assessment during routine monitoring, guiding the treatment before the outcome worsens. In individuals with persistent elevation of ctDNA, local therapy is associated with an increased relapse rate while monitoring for the presence of residual disease, which could theoretically prevent or delay relapse [34]. However, the evidence that the outcome will improve with a change in treatment regimen with the help of detecting ctDNA has yet to be proved. The above facts were also stated by M Scarlotta et al. and Dang DK et al. in their respective studies.

A systematic review was done by Ocana A et al. on the ability of ctDNA to predict the prognosis and its association with OS. The assessments showed that a positive ctDNA assay would likely have worse OS, with an OR of 4.83 and a confidence interval of 95%. It stated that the OS would not change with the tumor site. These results are yet to be researched, and their reliability is unknown [35].

X Chen et al. conducted a second longitudinal follow-up study to determine the predictive utility of multi-cancer early detection (MCED) and its relation to OS with a follow-up period of three years involving 2129 patients, with only 21% of participants reaching the third year of follow-up. The study determined that OS was better with the cancers not detected by MCED (P<0.0001%), which points us to the fact that MCED could be used as a prognostic indicator. Furthermore, the study noted that cancers detected by routine screening tests had a better outcome when compared to the ones detected by MCED, which were more aggressive. It opposed the statement of an increased rate of overdiagnosis through MCED, as most of the standard screening tests have overdiagnosed cancer, with a rate ranging from 25% to 60%, depending on the type of cancer. Hence, the risk may not be as high as expected because of its preferential detection of clinically significant cancers. Before drawing any conclusions, lead time bias must be thoroughly assessed [36].

When OS, PFS, and six-month treatment response were considered in individuals after conducting a ctDNA assay, the concordance rate with tissue biopsy ranged from 69% to 90%, depending on the mutation detected and the time frame. Meanwhile, M Scarlotta et al. established the utility of post-operative ctDNA levels as a predictor for tumor recurrence. Fifteen out of 16 individuals with high ctDNA levels in the postop period developed recurrence. The correlation of ctDNA levels with tumor burden is more significant than the conventional marker CA15-3 for breast cancer. The levels were also correlated to the stage, grade, size of the tumor, and lymph node metastasis [38].

A relation between pre-operative levels and the outcome (recurrence and survival) was observed. The PFS and OS were shorter for patients with high preop levels, with a p-value of <0.0001%. The study assumed that the levels of ctDNA would be higher in patients with incomplete resection and occult metastasis. CtDNA offers both qualitative and quantitative analysis of disease progression, recurrence, and response to treatment and, hence, has an excellent potential for future implementation in clinical practice [39].

Advantages of CtDNA Over Conventional Screening Modalities

According to all the data presented in the selected articles, the ctDNA study is a blood-based analysis that is safe and non-invasive compared to other screening tests like mammography, LDCT, colonoscopy, and others. As the half-life of ctDNA is very short, it could provide real-time analysis of tumor characteristics, which could identify the new mutations, response to treatment, or disease progression, thereby guiding the treatment. The PCR could detect tumor-specific mutations concordant with those detected through tissue sampling, making targeted therapy possible. In contrast to tumor-specific screening tests, which require multiple visits for multiple cancers, a one-visit single blood draw technique would be of greater convenience. Analysis of ctDNA in the pre- and post-operative evaluation could be used as a marker to predict the outcome of surgical intervention and to estimate the OS and PFS. The cost of ctDNA assay was estimated to be around 500$ by JD Cohen et al., which is low compared to other screening tests like colonoscopy and could be cost-effective when implemented as a single screening test for multicancer detection.

Limitations of CtDNA in Clinical Practice

Certain limitations of ctDNA have to be addressed before implementation in clinical practice. First, according to the calculations provided by C Fiala et al. using the example of the fetus as a tumor growing in the mother, when the level of tumor DNA is below 0.01% (one tumor DNA for 10,000 normal DNA), it could not be detected by the conventional 10mL blood draw sample (equivalent to 4 mL plasma sample). For the screening test to be considered ideal, the tumor size should not be greater than 5 mm (a tumor size of <5 mm is more likely to be localized, minimally progressive, and asymptomatic). In this scenario, the tumor DNA will be less than 0.01% compared to standard DNA, making it impossible to detect by the conventional blood-drawn sample of 10 mL containing 4 mL of plasma. The assay could only detect a tumor size above 1 cm, usually symptomatic or detected by other radiographic techniques [41].

The clinical validity and utility still need to be established. Higher false positive rates accounting for clonal hematopoiesis are a significant challenge in the assay, which could pose increased risks of anxiety in patients and increase the cost of detecting an unknown source. Furthermore, the assay could not identify the tumor's location, which a set of studies for localization and confirmation should follow up. The levels of ctDNA may vary depending on the tumor type, location, and sampling errors. Hence, setting a cut-off value for considering the test to be positive has yet to be determined. The frequency of screening and surveillance testing is not stated in any articles. The sensitivity and specificity of the study are yet to be confirmed by conducting large amounts of clinical studies for the assay to be approved for screening and are reliable only with advanced tumors. There is a considerable drop in the values while testing for early-stage tumors. As estimated by Abbosh et al., the cost for performing a complete personalized assay would be around 1750$. The tests require trained personnel to interpret the results and are very time-consuming compared to other testing strategies. As expected, the assay may sometimes detect different mutations than tissue samples.

Strengths and Limitations of Our Study

No RCTs were available, and only a few observational studies could be reviewed, which is a major limitation of the study. This is only a review article and may not represent the statistics accurately. Potential bias might not be considered while analyzing the articles. The study may have included only some of the data available and could have missed the articles while searching the database. However, this review presented detailed findings of the available studies in a simplified and understandable fashion. Our review combined most of the available data on ctDNA as a screening, prognostic, and surveillance tool into a single study with all the required information.

## Conclusions

After an extensive review of the collected articles, it is concluded that the role of ctDNA in early cancer detection has not proven helpful. Even if the test could detect the mutations, an extensive workup for the search of tumor is required as the assay could only detect but cannot localize the disease. However, due to its specific feature of recognizing the tumor-related mutations, it could be implemented as a supplemental tool to assess the nature of the tumor, grade, and size of the tumor and for predicting the outcomes of the tumor, such as progression rate, metastasis, response to treatment, resistant mutations by pre-operative and post-operative evaluation of the tumor marker, ctDNA and thereby estimating PFS and OS depending on the level of marker present. Much research is required to determine the sensitivity, specificity, false positive rates, and false negative rates to determine the true potential as a screening tool, which was limited by huge research costs. The clinical validity and utility must be established if ctDNA has to be implemented in future clinical practice.
